# Chenodeoxycholic Acid from Bile Inhibits Influenza A Virus Replication via Blocking Nuclear Export of Viral Ribonucleoprotein Complexes

**DOI:** 10.3390/molecules23123315

**Published:** 2018-12-14

**Authors:** Ling Luo, Weili Han, Jinyan Du, Xia Yang, Mubing Duan, Chenggang Xu, Zhenling Zeng, Weisan Chen, Jianxin Chen

**Affiliations:** 1 Guangdong Provincial Key Laboratory of Veterinary Pharmaceutics Development and Safety Evaluation, College of Veterinary Medicine, South China Agricultural University, Guangzhou 510642, China; lingluo@stu.scau.edu.cn (L.L.); dujinyan2014@163.com (J.D.); m15902070514@163.com (X.Y.); chgangxu@scau.edu.cn (C.X.); zlzeng@scau.edu.cn (Z.Z.); 2 Hygiene Detection Center, School of Public Health and Tropical Medicine, Southern Medical University, Guangzhou 510515, China; Picese.14@gmail.com; 3 Department of Biochemistry and Genetics, La Trobe Institute for Molecular Science, La Trobe University, Melbourne, VIC 3086, Australia; e.duan@latrobe.edu.au

**Keywords:** chenodeoxycholic acid (CDCA), influenza A virus, A549 cells, MDCK cells, nuclear export of viral ribonucleoprotein (vRNP)

## Abstract

Influenza A virus (IAV) infection is still a major global threat for humans, especially for the risk groups: young children and the elderly. The currently licensed antiviral drugs target viral factors and are prone to viral resistance. In recent years, a few endogenous small molecules from host, such as estradiol and omega-3 polyunsaturated fatty acid (PUFA)-derived lipid mediator protection D1 (PD1), were demonstrated to be capable of inhibiting IAV infection. Chenodeoxycholic acid (CDCA), one of the main primary bile acids, is synthesized from cholesterol in the liver and classically functions in emulsification and absorption of dietary fats. Clinically, CDCA has been used in the treatment of patients with cholesterol gallstones for more than five decades. In this study, we showed that CDCA attenuated the replication of three subtypes of influenza A virus, including a highly pathogenic H5N1 strain, in A549 and MDCK cell cultures with IC_50_ ranging from 5.5 to 11.5 μM. Mechanistically, CDCA effectively restrained the nuclear export of viral ribonucleoprotein (vRNP) complexes. In conclusion, as an endogenous physiological small molecule, CDCA can inhibit IAV replication in vitro, at least in part, by blocking vRNP nuclear export, and affords further studies for development as a potential antiviral agent against IAV infections.

## 1. Introduction

Influenza A viruses (IAVs) and their continuous re-emergence are a major global concern. The morbidity and mortality and the socioeconomic burden associated with IAV infection remain very significant. Seasonal IAV infection is responsible for up to half a million deaths worldwide. However, such a number could significantly increase during pandemics that occurred every 10 to 50 years [1]. The highly pathogenic avian influenza virus H5N1 in 1997 and 2003, H7N7 in 2003, H1N1 pandemic strain in 2009 (H1N1pdm09), and H7N9 in 2013—which showed very high mortality in the affected populations [2,3,4]. Available vaccines only protect against antigenically related strains with limited efficacy, especially in the elderly, a population at higher risk of severe influenza. While antiviral drugs are mostly used either prophylactically or in treatment, they have minimal impact on the course of the disease. Heavy reliance on antiviral drugs has also placed a strong selective pressure on IAVs to mutate and develop resistance, as is the case with the viral neuraminidase inhibitor oseltamivir [5,6].

A promising approach for discovery of therapeutic compounds is to target host factors that regulate viral replication. In this respect, genomics and proteomics studies have given us abundant information on host genes and proteins associated with viral infection and pathogenesis [7]. It has been reported that host cells produce many proteins—such as IFITM3, ISG15, MxA, viperin, tetherin, HDAC6, and MCPIP1—that have the natural ability to restrict influenza virus infection [8,9,10]. Additionally, Morita et al. reported that the omega-3 polyunsaturated fatty acid (PUFA)-derived lipid mediator protectin D1 (PD1) markedly attenuated IAV replication via RNA export machinery; and PD1 treatment improved the survival and reduced pathology induced by lethal IAV infection in mice [11]. Among all host factors involved in viral replication and/or pathogenesis, it is likely that some of them could be targeted for the treatment of IAV infection without significant side effects.

The primary bile acids—cholic acid (CA) and chenodeoxycholic acid (CDCA)—are synthesized in the liver from cholesterol and classically function to emulsify and absorb dietary fats and lipid-soluble vitamins. Primary bile acids are transformed by intestinal bacteria into secondary bile acids, deoxycholic acid (DCA), lithocholic acid (LCA), and ursodeoxycholic acid (UDCA) [12]. While the secreted bile acids travel through the intestine, they are reabsorbed in the ileum and return to the liver via the portal vein [13]. This enterohepatic circulation is essential in maintaining an effective concentration of bile acids and cholesterol homeostasis. Clinically, CDCA has been used in the treatment of patients with cholesterol gallstones since the 1970s [14]. Recently, CDCA was shown to have inhibitory activities against some viruses specifically infecting organs of the digestive system including rotavirus, hepatitis B, and D virus [15,16]. As many antiviral compounds often have the ability to inhibit different viruses and as CDCA is a natural product, we wondered whether CDCA also possess antiviral activity against IAV infection as this had not been reported.

In this study, we examined the antiviral effects of CDCA against IAVs in vitro. We found that CDCA efficiently inhibited the replication of H5N1, H9N2, and H1N1 viruses in A549 and MDCK cell cultures. Furthermore, we demonstrated that CDCA inhibited IAV replication by restraining the nuclear export of vRNP complexes. To the best of our knowledge, this is the first report on CDCA’s anti-IAV activity.

## 2. Results

### 2.1. CDCA Inhibited IAV Replication in A549 and MDCK Cells

#### 2.1.1. Cell Cytotoxicity of CDCA

**Figure 1:**
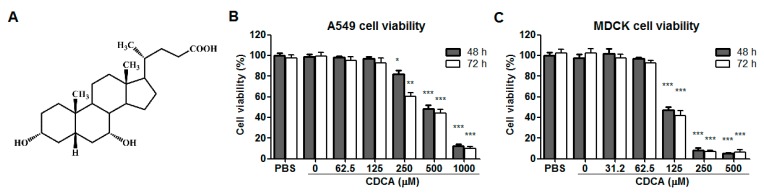
Chemical structure and cytotoxicity of chenodeoxycholic acid (CDCA). (**A**) Chemical structure of CDCA. (**B**) Cytotoxicity of CDCA on A549 cells. (**C**) Cytotoxicity of CDCA on MDCK cells. Confluent A549 or MDCK cells were treated with different concentrations of CDCA. After 48 h or 72 h, cell viability was measured by 3-(4,5-dimethylthiozol-2-yl)-3,5-dipheryl tetrazolium bromide (MTT) assay. The results are expressed as percentage (%) of mock treated cells (represented with PBS). Values represent the mean (%) ± SD from 3 independent determinations. * *p* < 0.05, ** *p* < 0.01 and *** *p* < 0.001 compared to the PBS control.

Cell cytotoxicity of CDCA (chemical structure shown in Figure 1A) was first determined in A549 and MDCK cells using the 3-(4,5-dimethylthiozol-2-yl)-3,5-dipheryl tetrazolium bromide (MTT) assay. As shown in Figure 1B,C, no obvious cytotoxicity was observed for CDCA concentrations ≤125 μM on A549 cells and ≤62.5 μM on MDCK cells, respectively, after 48 or 72 h treatment. Thus, 60 and 120 μM of CDCA were selected as the maximum concentrations for further studies using these cell lines. The CC_50_ levels (the concentration required to reduced normal cell viability by 50%) of CDCA were 372.5 μM on A549 cells and 121.2 μM on MDCK cells (Table 1), respectively.

#### 2.1.2. CDCA Protected Cells from IAV Infection

**Figure 2:**
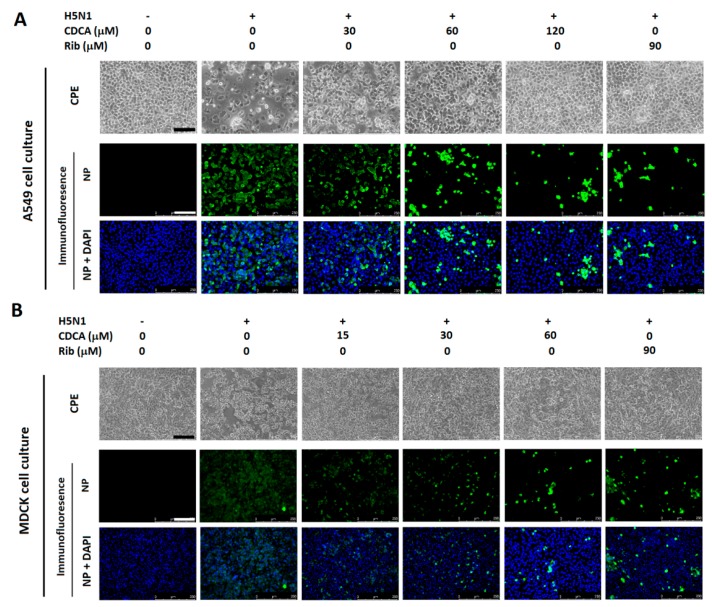
CDCA inhibited H5N1 replication in A549 and MDCK cell cultures. A549 (**A**) or MDCK (**B**) cells grown in 96-well plates were infected with H5N1 virus (0.1 MOI) for 1 h at 37 °C and then cultured in fresh medium containing various concentrations of CDCA. At 48 hpi, cytopathic effect (CPE) was observed under microscopy (upper panels of **A** and **B**). Then the cells were fixed with paraformaldehyde and the viral NP expression was detected by indirect immunofluorescence assay (IFA). Representative IFA images for A549 (**A**) and MDCK (**B**) cells from three independent experiments were shown. Scale bar: 125 µm.

Next, we examined the protective effects of CDCA on A549 and MDCK cells from highly pathogenic H5N1 virus infection via observing cytopathic effect (CPE) and virus distribution profile reflected by viral nucleoprotein (NP) expression using immunofluorescence microscopy at 48 hpi. As shown in Figure 2A,B, H5N1 replication was significantly inhibited and cells were protected by CDCA in a dose-dependent manner. Ribavirin, a well-known inhibitor of viral RNA synthesis [17], was used as a positive antiviral drug control in this study. Our results showed that 90 μM of ribavirin exhibited a significant inhibition on H5N1 infection in the same assays.

**Figure 3:**
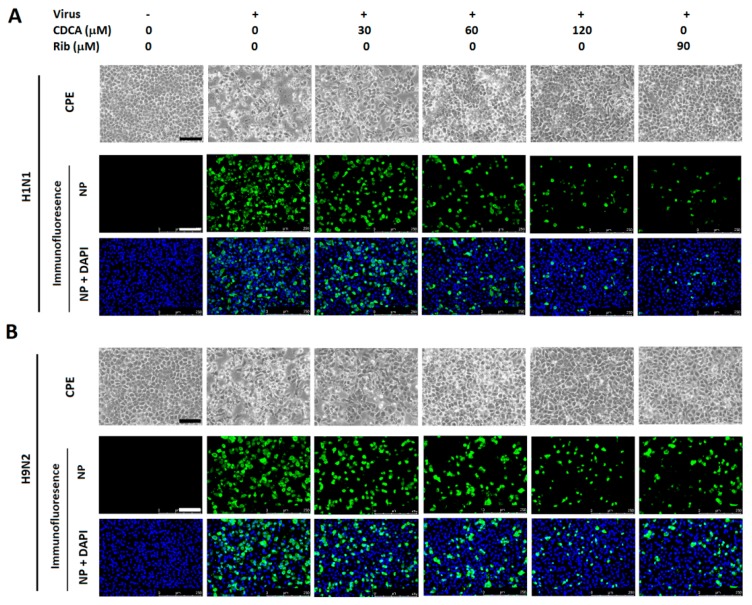
CDCA inhibited PR8 (H1N1) and H9N2 replication in A549 cell cultures. Cells grown in 96-well plates were infected with PR8 (H1N1) (**A**) or H9N2 (**B**) virus (0.5 MOI) for 2 h at 37 °C and then cultured in fresh medium containing various concentrations of CDCA. At 48 hpi, CPE was observed under microscopy (upper panels of **A** and **B**). Then the cells were fixed with paraformaldehyde and the virus NP expression was detected by IFA as described in Figure 2. Scale bar: 125 µm.

To explore whether CDCA possesses broad inhibitions on various IAV subtypes, two other representative subtypes, classic PR8 (H1N1) and H9N2 virus, were investigated using immunofluorescence microscopy. As shown in Figure 3A,B, CDCA exhibited anti-IAV activities reduced CPE in A549 cells in a dose-dependent manner and against both viruses assessed at 48 hpi, similar to the inhibition observed on H5N1 virus, indicating CDCA’s IAV inhibition is subtype independent.

#### 2.1.3. CDCA Inhibited Production of Progeny Virus

**Figure 4:**
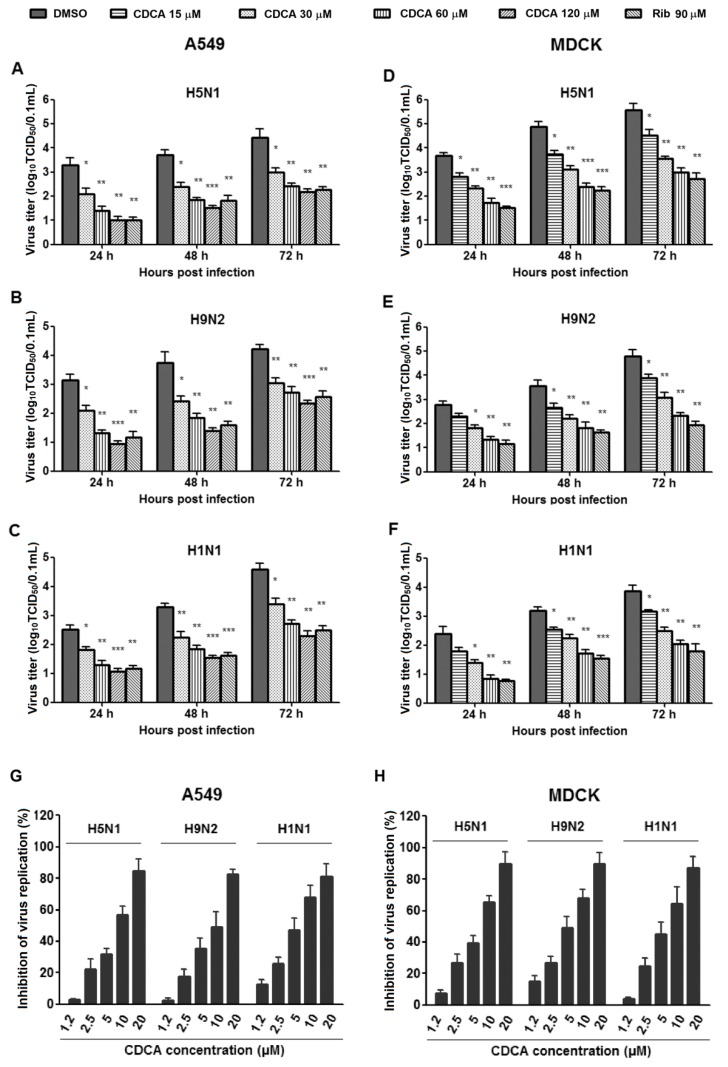
CDCA reduced progeny influenza A virus (IAV) yield in A549 and MDCK cell cultures. A549 or MDCK cells grown in 24-well plates were infected with influenza virus H5N1 (0.1 MOI) for 1 h, or H9N2 (0.5 MOI) or PR8 (H1N1) (0.5 MOI) for 2 h, respectively, and then cultured in fresh medium containing various concentrations of CDCA. At indicated timepoints postinfection, cells and supernatants were collected and subjected to viral titer determination using the end point dilution assay. Virus titer was expressed as log_10_ TCID_50_/0.1 mL. (**A**–**C**) CDCA inhibited replication of H5N1, H9N2, and H1N1 viruses in A549 cell cultures, respectively. (**D**–**F**) CDCA inhibited replication of H5N1, H9N2, and H1N1 viruses in MDCK cell cultures, respectively. Data are presented as mean ± SD of results from three independent experiments. * *p* < 0.05, ** *p* < 0.01 and *** *p* < 0.001 compared to the respective virus control (represented with DMSO). (**G**,**H**) CDCA reduced IAV progeny yield in a dose-dependent manner at 48 hpi. The percent inhibition of virus replication was calculated as a ratio of reduced virus titer from a CDCA-treated sample (compared with the DMSO control) to virus titer from the DMSO control.

We further studied the IAV inhibition kinetics by CDCA at various concentrations. For the IAV-infected control, virus titers increased persistently from 12 to 72 hpi. The treatment of CDCA significantly inhibited progeny virus titers in a dose-dependent manner at all timepoints, both in A549 and MDCK cells as shown in Figure 4. Interestingly, CDCA exhibited similar inhibition kinetics against three different subtype IAV strains. The addition of 90 μM ribavirin resulted in a reduction of progeny virus production similar to the treatment results of 60 μM CDCA in A549 cells, although slightly more potent in MDCK cells.

The significantly inhibitory activities of CDCA on IAV infections in vitro lead us to wonder whether other two bile acids hyodeoxycholic acid (HDCA) and ursodeoxycholic acid (UDCA), which are structural isomers of CDCA (Figure S1), were also able to inhibit IAV replication. We therefore determined the inhibitory activities of HDCA and UDCA on the three IAV (H5N1, H9N2, and H1N1) infections in A549 and MDCK cells at various concentrations at 48 hpi using an in vitro virus growth inhibition assay described in the methods. The IC_50_ values (concentration of compound required to inhibit IAV titer by 50%) of HDCA, UDCA, and CDCA were calculated based on the dose–inhibition curves (Figure 4G,H). As shown in Table 1, CDCA again showed significant inhibition on the three virus infections with IC_50_ ranging from 5.5 to 11.5 μM, while HDCA and UDCA showed much weaker inhibition with IC_50_ ranging from 31.0 to 73.7 μM.

### 2.2. CDCA Interfered with the Later Stage of the Virus Lifecycle

**Figure 5:**
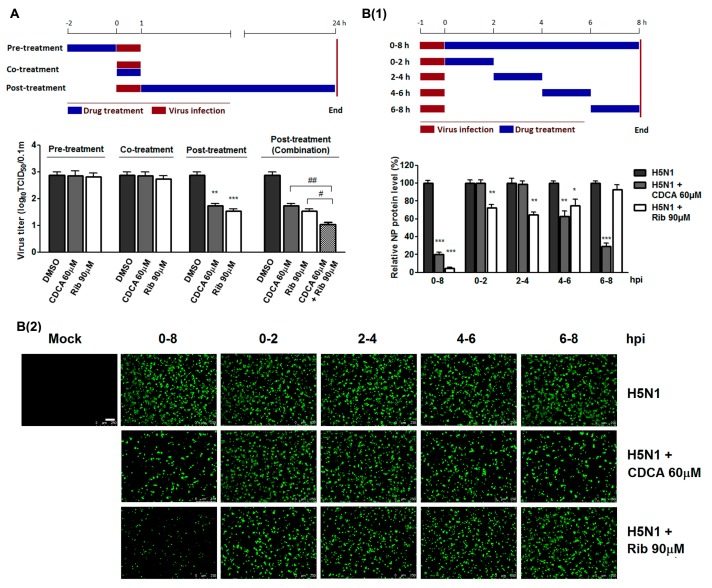
CDCA inhibited H5N1 replication by targeting the later stage of the virus lifecycle. (**A**) Time course inhibition assay. A549 cells grown in 24-well plates were treated with CDCA or ribavirin or their combination for 2 h prior to virus infection (pretreatment), for 1 h during the viral adsorption period (cotreatment), or for 24 h after 1 h virus infection and removal (post-treatment). For three treatment models, 0.1 MOI of H5N1 was used for infecting cells for 1 h. At 24 hpi, supernatants were collected for determining virus titer using the end point dilution assay. Upper panel: the experimental design. Lower panel: virus titers from three independent experiments. (**B**) Time-of-addition assay. A549 cells grown in 24-well plates were infected with 1.0 MOI of H5N1 for 1 h. After two washes with PBS, 60 µM of CDCA or ribavirin was added at the indicated times and removed after 2 h or 8 h. After each incubation period, the cells were incubated with fresh medium until 8 hpi. The cells were then subjected to viral NP protein analysis using IFA. The experimental design was shown in the upper panel (**B1**). Results shown in the lower panel (**B1**) are normalized NP protein levels based on the fluorescence optical densities (OD) of the images from three independent experiments. Software Image J was used to digitize image OD; results from CDCA or ribavirin treated samples were compared to those from corresponding DMSO-treated control groups (set as 100%). Representative IFA images of the three independent experiments are shown in the **B2** panel. Scale bar: 125 µm. Statistical significances are denoted by * *p* < 0.05, ** *p* < 0.01, and *** *p* < 0.001 compared to DMSO control, and ^#^ *p* < 0.05 and ^##^ *p* < 0.01 between indicated groups.

To identify the stage(s) of the influenza lifecycle during which CDCA plays its inhibitory role, we performed time course studies of the inhibitory effects of 60 μM CDCA. As shown in Figure 5A and Figure S2, CDCA pretreatment did not reduce progeny virus yield and viral NP production, indicating that CDCA did not impair the susceptibility of A549 cells to IAV. CDCA cotreatment for the first hour did not affect virus replication either, indicating that CDCA did not directly interact with IAV and block virus binding to and entering the cells. When the cells were treated with CDCA for 24 h post IAV infection (post-treatment), progeny virus yield decreased more than 90%, which was also reflected by the decreased viral NP production (Figure S2), indicating that CDCA exerted antiviral effects during stages post IAV entry. Interestingly, the combination of 60 μM of CDCA and 90 μM of ribavirin resulted in more decrease of progeny virus titer than that when either was used alone, suggesting an additive or synergetic antiviral effect of CDCA and ribavirin against IAV replication (Figure 5A).

It has been reported that the time from entry into the cell to the production of new virus particles is on average 6–8 h, depending on cell type [18]. To identify the exact stage(s) of IAV lifecycle affected by CDCA, we chose five time intervals (0–2, 2–4, 4–6, 6–8, and 0–8 h) to investigate the time course inhibition via assessing NP expression. As shown in Figure 5(B1(low panel),B2), a more significant inhibition of virus replication, as represented by NP expression, was observed when CDCA was added to the A549 cells within the time intervals of 4–6 and 6–8 hpi, when compared to the earlier time intervals before 4 hpi, indicating that CDCA exerted effects during the late stages of IAV infection, i.e., vRNP nuclear export and assembly. As expected, because ribavirin inhibits viral RNA synthesis, it facilitated much better IAV inhibition when added to the infected A549 cells before 6 hpi. It showed no inhibition when added during the late infection stage of 6–8 hpi.

### 2.3. CDCA Blocked the Nuclear Export of Viral RNP Complexes

**Figure 6:**
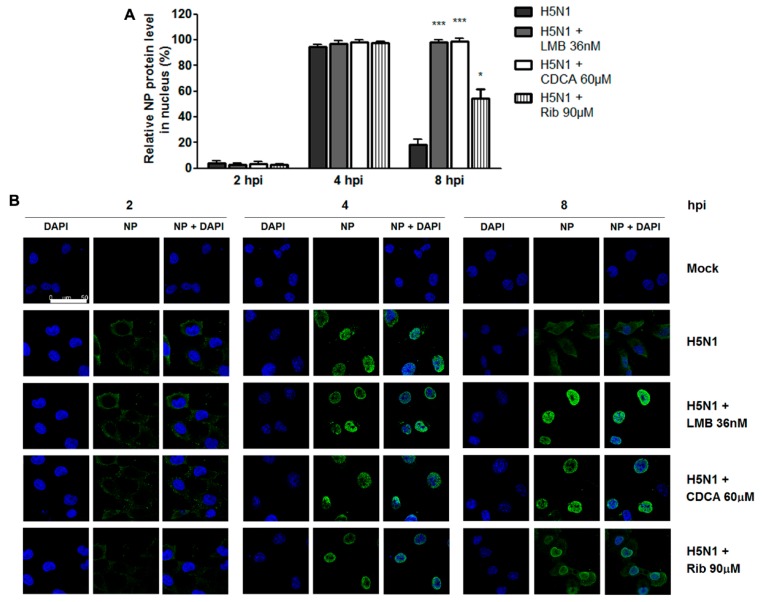
CDCA blocked viral RNP nuclear export. A549 cells were infected with H5N1 (1 MOI) for 1 h. After two washes with PBS, the cells were incubated in fresh medium containing CDCA or ribavirin or Leptomycin B (LMB). At the indicated timepoints, the cells were fixed and viral ribonucleoprotein (RNP) localization was determined by immunofluorescence staining. Cells were imaged using a confocal laser scanning microscope. Fluorescence optical densities in the whole cells or their nuclei were quantitated using software Image J. Results shown in (**A**) are the ratios of NP protein levels in the nuclei to that of the whole cells based on the fluorescence optical densities of the images from three independent experiments. Representative confocal IFA images out of three independent experiments were shown in (**B**). Scale bar: 50 µm. Statistical significances are denoted by * *p* < 0.05 and *** *p* < 0.001 compared to corresponding DMSO-control (H5N1).

During IAV infection, the nuclear export of viral ribonucleoproteins (vRNPs) is required for the final assembly of progeny virions before their release from the infected host cell [19]. To investigate the effect of CDCA on IAV RNP nuclear export, we observed the dynamic change of H5N1 RNP in the cytoplasm and nucleus (reflected by NP staining) in the presence or absence of CDCA between 0 to 8 hpi. As shown in Figure 6, newly synthesized NP proteins were predominantly localized in the cytosol at 2 hpi and then translocated to nucleus at 2 and 4 hpi. The presence of CDCA did not affect such early events (Figure 6). At 8 hpi, the vRNP complexes were almost completely exported from the nucleus in the H5N1-infected cells. However, Leptomycin B (LMB)—an inhibitor of the nuclear export receptor CRM1—as a positive control, efficiently blocked the nuclear export of vRNPs. Notably, the nuclear export of vRNP complexes was also inhibited by CDCA. Interestingly, although ribavirin did not block the nuclear export of vRNPs, it looked likely to put off this process. We speculate that the reduction of vRNP nuclear export in the ribavirin-treated cells might be due to reduced vRAN replication and delayed vRNP assembly as a result of RNA polymerase inhibition by ribavirin. Taken together, CDCA inhibits infection of IAV by blocking nuclear export of its vRNP complexes.

## 3. Discussion

Chenodeoxycholic acid (CDCA), one of the most abundant primary bile acids, is synthesized from cholesterol in the liver and functions to emulsify and absorb dietary fat and lipid-soluble vitamins [12]. Clinically, CDCA has been used in the treatment of patients with cholesterol gallstones for more than 50 years [14]. In the present study, our findings revealed that CDCA exhibited potent inhibitory activities against three different IAV strains including a high pathogenic H5N1 virus in vitro. CDCA treatment resulted in a remarkable decrease of progeny viruses both in infected A549 and MDCK cells. Interestingly, two CDCA-derived secondary bile acids ursodeoxycholic acid (UCDA) and hyodeoxycholic acid (HDCA) exhibited much weaker inhibitory activities against IAV. Structurally, UDCA is an epimer and HDCA is an isomer of CDCA, and the three compounds share the same steroid nucleus skeleton. Comparing the chemical structures (Figure S1) and anti IAV activities of the three compounds (Table 1), it can be concluded that the alpha oriented hydroxyl group in the 7-C position of the B ring of CDCA is important for its potent anti-IAV activities.

One IAV lifecycle is orderly composed of virus binding, internalization, and RNA replication and viral protein synthesis, assembly, budding, and release from infected cells. The vRNP complexes of IAV are composed of viral RNA (vRNA), NP and three vRNA-dependent RNA polymerase subunits PA, PB1, and PB2. They are assembled in the nucleus and must be exported to the cytoplasm during the late stages of infection, which is crucial for productive viral infection [20]. It has been illustrated that vRNP nuclear export starts at 4–6 hpi and continues for several hours (the actual time depends on the cell line involved) [21,22,23]. Through the time course inhibition experiments (Figure 5B), we found that CDCA exerted inhibition on IAV replication when it was added within the time intervals of 4–6 & 6–8 hpi, later stages of the virus life cycle. Furthermore, we showed that CDCA potently blocked vRNP complexes nuclear export at 8 hpi by observing dynamic vRNP translocation post H5N1 infection (Figure 6), which was consistent with our observations made from the time course inhibition experiment (Figure 5B). We speculate that the property of CDCA blocking vRNP nuclear export might be closely related to its effect on impairing IAV replication in vitro. This is a significant finding as other inhibitors affect IAV differently, such as ribavirin that directly interacts with IAV polymerase. It is therefore possible to combine the CDCA and other IAV inhibitors to achieve synergistic effect.

Yunjeong et al. demonstrated that CDCA significantly reduced rotavirus replication both in vitro and in vivo. The inhibition mechanism was proposed to be downregulating lipid synthesis induced by rotavirus infection [15]. Yan et al. identified sodium taurocholate cotransporting polypeptide (NTCP) as a cellular receptor for human hepatitis B virus (HBV) and hepatitis D virus (HDV) viral entry and found that some bile salts including CDCA were capable of inhibiting HBV and HDV infection mediated by NTCP [16]. In this study, we showed that CDCA inhibited IAV replication by blocking vRNP nuclear export, a novel antiviral mechanism compared to those of licensed IAV inhibitors.

Export of vRNPs to the cytosol occurs late in IAV infection. It has been confirmed that M1 is essential for vRNP export from the nucleus to the cytosol [24]. The influenza virus NS2 protein (also called nuclear export protein, or NEP) is responsible for vRNP nuclear export via its interaction with M1 and a cellular protein called CRM1 [25]. In addition, during productive virus infection the proapoptotic factors TNF-related apoptosis inducing ligand (TRAIL), Fas, and Fas-L are expressed in an NF-κB-dependent manner. These factors induce caspase activation and subsequent apoptosis [26]. Faleiro and Lazebnik showed that active caspases directly or indirectly increased the diffusion limit of nuclear pores, allowing vRNPs to leave the nucleus by diffusion during apoptosis [27]. Furthermore, Wurzer and coworkers showed that apoptosis induction in IAV infected cells, in particular activation of caspase-3, was a prerequisite for efficient vRNP migration out of the nucleus, and inhibition of caspase-3 activity resulted in nuclear vRNP retention [28]. The anti-apoptosis effect of CDCA has been well established by previous studies. For instance, Lian and coworkers demonstrated that CDCA, as a FXR agonist, exerts an anti-apoptotic role in a concanavalin A induced autoimmune hepatitis mouse model via downregulating Fas/Fas ligand, TRAIL, and caspase-3 [29]. Similarly, Hirano and coworkers showed that CDCA inhibited apoptosis through inducing cIAP-1 (cellular inhibitor of apoptosis protein-1) expression in hepatocytes [30]. In this study, we showed that CDCA blocked vRNP nuclear export during high pathogenic (HP) H5N1 virus infection. HP H5N1 virus has been reported to be more potent to induce infected cells to undergo apoptosis [31]. We therefore speculate that the inhibition of CDCA on vRNP nuclear export might be closely related to its effects on alleviating apoptosis via downregulating Fas/Fas-L, TRAIL and/or caspase-3. Alternatively, it is possible that CDCA might directly interact with either M1 or NS2 to affect vRNP nuclear export. In either case, further studies are required to elucidate the detailed mechanisms on how CDCA blocks nuclear export of vRNP complexes.

In conclusion, CDCA is a physiological product and has been licensed clinically as cholelitholytic agent for decades. Our data demonstrated that CDCA effectively inhibited IAV replication in A549 and MDCK cells. Mechanistically, CDCA inhibited IAV replication via blocking vRNA nuclear export. CDCA may potentially be used as a novel strategy to treat a severe or pandemic influenza infection and necessitates further studies, especially its in vivo antiviral activity.

## 4. Materials and Methods

### 4.1. Cell Lines and Virus Strains

A549 (human lung carcinoma) and MDCK cells (Madin–Darby canine kidney cells) were obtained from the Center of Cellular Resource, Chinese Academy of Sciences (Shanghai, China). All cells were grown at 37 °C in Dulbecco’s Modified Eagle’s Medium (DMEM, Gibco, UT, USA) supplemented with 10% fetal bovine serum (FBS, Biological Industries, Kibbutz Beit Haemek, Israel), 100 U/mL of penicillin, and 100 μg/mL streptomycin in a humidified atmosphere with 5% CO_2_.

The H1N1 IAV strain A/Puerto Rico/8/34 (PR8) virus was obtained from the Chinese Center for Disease Control and Prevention (Beijing, China). Avian IAV strains A/Duck/Guangdong/99 (H5N1) virus and A/Chicken/Guangdong/96 (H9N2) were kindly provided by the Veterinary Technology Center of South China Agricultural University (Guangzhou, China). Virus stocks were passaged in 10-day old embryonated chicken eggs for 48 h. The allantoic fluid was harvested and aliquots were stored at −80°C until required. Viral titers were determined as the 50% tissue culture infectious dose (TCID_50_/0.1mL) in confluent MDCK cells in 96-well microtiter plates using the endpoint dilution assay as described previously [32]. Experiments involving H5N1 virus strains were conducted in a physical containment level three (PC3) laboratory.

### 4.2. Compounds

Chenodeoxycholic acid (CDCA), hyodeoxycholic acid (HDCA), and ursodeoxycholic acid (UDCA) were purchased from Sichuan Victory Biotechnology Co. Ltd. (Chengdu, China) with HPLC purities ≥98%. Ribavirin hydrochloride was purchased from GuangDong Starlake Bioscience Co. Ltd. (Zhaoqing, China) with purities of HPLC ≥99%. Leptomycin B (LMB) was purchased from Santa Cruz Biotechnology (Dallas, TX, USA). Drugs were dissolved in dimethyl sulfoxide (DMSO) and diluted with PBS to <0.4% DMSO.

### 4.3. Cytotoxicity Assay

The cytotoxicity of tested compounds was evaluated using MTT assay [18]. Briefly, cells were grown in 96-well plates for 24 h. The medium was replaced with fresh medium containing serially diluted compounds and the cells were further incubated for 48 h or 72 h. The culture medium was removed and replaced with 100 μL 3-(4,5-dimethylthiozol-2-yl)-3,5-dipheryl tetrazolium bromide (MTT; Sigma-Aldrich, MA, USA) solution (1 mg/mL in PBS) and incubated at 37 °C for 4 h. After removal of the supernatant, 150 μL of DMSO was added to all of the wells to dissolve the formazan crystals for 15 min at 37 °C Cell viability was then measured as the absorbance at 490 nm with a microplate reader (Thermo fisher scientific, MA, USA) and expressed as a percentage of the control level. The mean optical density (OD) values from six replicated wells per treatment were used as the cell viability index. The 50% cytotoxic concentration (CC_50_) was analyzed by GraphPad Prism 5.0 (GraphPad Software, San Diego, CA, USA).

### 4.4. Indirect Immunofluorescence Assay (IFA)

Indirect immunofluorescence assay was used to perform a rapid evaluation of antiviral activities of compounds against IAV infection. For immunostaining, the IAV-infected or control cells were fixed with 4% paraformaldehyde for 10 min, then permeabilized with 0.25% Triton X-100 for 10 min at room temperature (RT). Cells were blocked with 1% bovine serum albumin (BSA) for 60 min at RT and then incubated with a mouse monoclonal antibody against influenza nucleoprotein (1:500 dilution, Sino Biological, Beijing, China) at 4 °C overnight. After three washes with PBS, the cells were incubated for 1 h at RT with an anti-mouse IgG antibody conjugated with Alexa Fluor^®^ 488 (green) (Cell Signaling Technology, MA, USA) at 1:1000 dilution. Nuclei were counterstained using 50 μL of 4,6-diamidino-2-phenylindole (DAPI, 300 nM; Sigma-Aldrich, MA, USA). Immunofluorescence was captured using the Leica DMI 4000B fluorescence microscope (Leica, Wetzlar, Germany).

### 4.5. In Vitro Virus Growth Inhibition Assay

A549 and MDCK monolayers were infected with virus for 1 h (H5N1) or 2 h (H9N2 and H1N1), respectively. Supernatants were removed and cells then incubated with DMEM containing serial concentrations of tested compounds. Cells and supernatants were collected at indicated timepoints post-infection and in total subjected to three freeze-thaw cycles at −80 °C and 4 °C to ensure maximal release of cellular virions [32,33]. Final supernatant viral titers were determined by the end point dilution assay using MDCK cells and expressed as log_10_ TCID_50_/0.1 mL [34]. The IC_50_ value (concentration of compound required to inhibit progeny viral titer by 50%) was determined by plotting the % inhibition of progeny viral titers as a function of compound concentration through a nonlinear regression analysis using the GraphPad Prism 5.0 software (GraphPad Software, San Diego, CA, USA).

### 4.6. Time Course Inhibition Assay

To estimate the influence of CDCA on IVA replication cycle, A549 cells were grown in 24-well plates to confluence and infected with H5N1 IAV (0.1 MOI) for 1 h at 37 °C CDCA or ribavirin or their combination was added before, during or after H5N1 infection. For pretreatment, cells were incubated with the indicated compounds for 2 h at 37 °C, followed by three washes with PBS and then infected with H5N1 for 1 h. For cotreatment, cells were simultaneously incubated with H5N1 and the compounds. After 1 h, the virus–drug mixture was removed and the cells were washed three times with PBS before fresh medium was added. For post-treatment, cells were first infected with H5N1 for 1 h followed by three washes with PBS and then incubated with medium containing the compounds. At 24 hpi, viruses in the supernatants were determined by the end point dilution assay and the extent of IAV infection in the cells was assessed by IFA for NP protein, respectively.

To determine the specific stage(s) of the viral life cycle affected by CDCA, a time-of-addition assay was performed as described by Liao et al. [18]. Briefly, confluent monolayers of A549 cells grown in 24-well plates were infected with H5N1 (1.0 MOI) for 1 h at 37 °C. Cells were washed three times with PBS to remove unbound viruses and incubated in fresh medium. 60 μM of CDCA or ribavirin was then added for 0 to 2, 2 to 4, 4 to 6, 6 to 8 h, or 0 to 8 hpi. After each incubation period, the cells were washed three times with PBS and incubated with fresh medium at 37 °C. At 8 hpi, the cells were subjected to viral NP protein analysis using IFA.

### 4.7. Viral RNP Nuclear Export Inhibition Assay

A549 cells grown on 10 mm cover slips (MatTek, MA, USA) were infected with H5N1 virus (1 MOI) for 1 h. The cells were washed three times with PBS and incubated with fresh medium containing 60 μM of CDCA or 36 nM of Leptomycin B (LMB) (an inhibitor of the nuclear export receptor CRM1, used as a positive control). At indicated timepoints, cells were washed with PBS, fixed with 4% paraformaldehyde in PBS for 10 min, then permeabilized with 0.25% Triton-X100 for 15 min, and blocked with 1% BSA in PBS for 30 min at RT. Cells were stained following the IFA protocol as described under 2.4. Cells were imaged using a TCS SP8 confocal laser scanning microscope (Leica, Wetzlar, Germany).

### 4.8. Statistical Analysis

All values are expressed as mean ± SD from at least three independent experiments. Statistical significance was determined by Student’s *t*-test when only two groups were compared or by one-way analysis of variance (ANOVA) when more than two groups were compared. Statistical analyses were performed using GraphPad Prism 6 (GraphPad Software, San Diego, CA, USA). * *p* < 0.05, ** *p* < 0.01, and *** *p* < 0.001 were considered to be statistically significant at different levels.

## Figures and Tables

**Table 1:** Cellular toxicity and inhibitory activity of CDCA, HDCA, and UDCA against influenza A virus replication in A549 and MDCK cells.

Drugs	CC_50_^a^ (μM)	H5N1		H9N2		PR8 (H1N1)	
		IC_50_^b^ (μM)	SI ^c^	IC_50_ (μM)	SI	IC_50_ (μM)	SI
On A549 cells							
CDCA	372.5 ± 41.2	8.2 ± 1.2	45.4	11.5 ± 1.7	32.4	7.7 ± 0.7	48.4
HDCA	485.7 ± 55.0	60.0 ± 5.5	8.1	53.0 ± 6.5	9.2	73.7 ± 6.7	6.6
UDCA	814.7 ± 49.2	34.2 ± 3.7	23.8	58.7 ± 4.5	13.9	43.0 ± 4.5	18.9
On MDCK cells							
CDCA	121.2 ± 9.7	6.5 ± 0.5	18.6	5.5 ± 0.7	22.0	7.2 ± 0.7	16.8
HDCA	413.5 ± 33.7	41.2 ± 3.5	10.0	31.0 ± 2.2	13.3	43.5 ± 2.7	9.5
UDCA	596.5 ± 53.0	47.2 ± 3.2	12.6	36.7 ± 3.5	13.3	56.2 ± 5.0	10.6

^a^ CC_50_, the concentration required to reduced normal, noninfected cell viability by 50%; ^b^ IC_50_, the concentration required to reduce the viral titer to 50%; ^c^ SI (selectivity index) is the ratio of CC_50_ to IC_50_. Data were presented as means ± SD of results from three independent experiments.

## Supplementary Materials

The Supplementary Materials are available online.
