# Supplementary material for: Chenodeoxycholic Acid from Bile Inhibits Influenza A Virus Replication via Blocking Nuclear Export of Viral Ribonucleoprotein Complexes
Source: Molecules. 2018 Dec 14;23(12):3315. doi: 10.3390/molecules23123315 (PMC6321071; doi:10.3390/molecules23123315)
Supplement: Supplementary file 1 [file molecules-23-03315-s001.pdf]

Supplementary figures

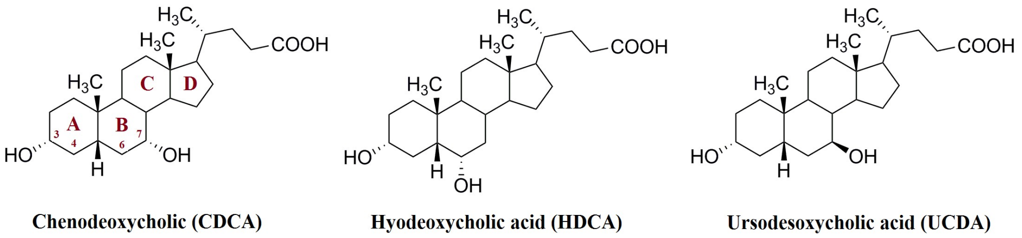

Figure S1. Chemical structures of CDCA, HDCA and UDCA.

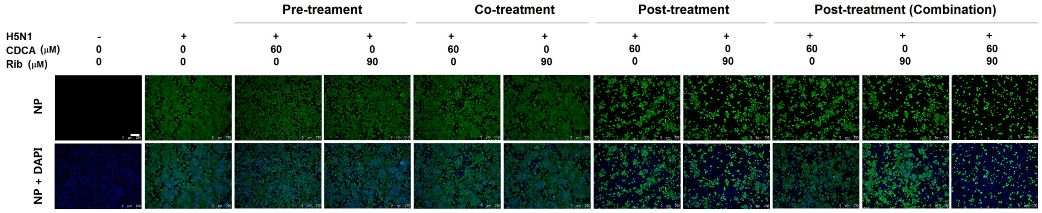

Figure S2. CDCA inhibited H5N1 replication in post-treatment model.

A549 cells grown in 24-well plates were treated with CDCA or ribavirin or their combination for 2 h prior to virus infection (pre-treatment), or for 1 h during the viral adsorption period (co-treatment), or for 24 h after 1 h virus infection and removal (post-treatment) (shown in Fig.5A). For three treatment models, 0.1 MOI of H5N1 was used for infecting cells for 1 h. At 24 hpi, supernatants were collected for virus titer determination using the end point dilution assay (Fig.5A), and the cells were stained for NP protein using IFA as described in the legend to Fig.2.
